# Leukocyte Redistribution: Effects of Beta Blockers in Patients with Chronic Heart Failure

**DOI:** 10.1371/journal.pone.0006411

**Published:** 2009-07-29

**Authors:** Stephan von Haehling, Joerg C. Schefold, Ewa Jankowska, Wolfram Doehner, Jochen Springer, Kristin Strohschein, Sabine Genth-Zotz, Hans-Dieter Volk, Philip Poole-Wilson, Stefan D. Anker

**Affiliations:** 1 Applied Cachexia Research, Dept. of Cardiology, Charité Medical School, Campus Virchow-Clinic, Berlin, Germany; 2 Department of Clinical Cardiology, National Heart & Lung Institute, Imperial College School of Medicine, London, United Kingdom; 3 Department of Nephrology and Intensive Care Medicine, Charité Medical School, Campus Virchow Clinic, Berlin, Germany; 4 Department of Cardiology, Military Hospital, Wroclaw, Poland; 5 Department of Medicine II, Johannes Gutenberg-University, Mainz, Germany; 6 Department of Medical Immunology, Charité Medical School, Campus Mitte, Berlin, Germany; University of British Columbia, Canada

## Abstract

**Background:**

Overproduction of pro-inflammatory cytokines is a well established factor in the progression of chronic heart failure (CHF). Changes in cellular immunity have not been widely studied, and the impact of standard medication is uncertain. Here we investigate whether a leukocyte redistribution occurs in CHF and whether this effect is influenced by beta-blocker therapy.

**Methodology:**

We prospectively studied 75 patients with systolic CHF (age: 68±11 years, left ventricular ejection fraction 32±11%, New York Heart Association class 2.5±0.7) and 20 age-matched healthy control subjects (age: 63±10 years). We measured the response of cells to endotoxin exposure *in vitro*, analysed subsets of lymphocytes using flow cytometry, and assessed plasma levels of the pro-inflammatory markers interleukin 1, 6, tumor necrosis factor-α, and soluble tumor necrosis factor receptors 1 and 2.

**Principal findings:**

While no differences in the number of leukocytes were noted between patients with CHF and healthy controls, we detected relative lymphopenia in patients with CHF (p<0.001 vs. control), mostly driven by reductions in T helper cells and B cells (both p<0.05). The number of neutrophils was increased (p<0.01). These effects were pronounced in patients who were beta-blocker naïve (32% of all patients with CHF). Increased plasma levels of soluble tumor necrosis receptor-1 correlated with the relative number of lymphocyte subsets.

**Conclusions:**

In patients with CHF, we detected a redistribution of leukocyte subsets, *i.e.* an increase in neutrophils with relative lymphopenia. These effects were pronounced in patients who were beta-blocker naïve. The underlying mechanism remains to be elucidated.

## Introduction

Chronic heart failure (CHF) is a multisystem disorder that affects not only the cardiovascular system but many other body organs and functions. The involvement of these systems can be explained by overproduction of neuroendocrine mediators and cytokines, which are a key mechanism for the progression of CHF. Elevated levels of catecholamines, among others, have long been recognized to play a role in this syndrome [1]. Both noradrenaline and adrenaline, released from nonsynaptic varicosities and the adrenal medulla, respectively, have profound effects on the immune system [2]. This may be of particular interest in heart failure, because evidence has accumulated over the last two decades to demonstrate convincingly that this disease represents a state of chronic inflammation [3]. Thus, modulation of inflammatory mediators and the cells from which they originate has become a focus of research in recent years [4]. Pro-inflammatory cytokines such as tumor necrosis factor-α (TNFα) and interleukin (IL) 1 and 6, have been shown to be independent predictors of poor survival in CHF [5], [6].

Few studies have investigated the interplay between pro-inflammatory cytokines and the redistribution of leukocyte subsets in patients with CHF and no studies have sought to elucidate the role of standard medical therapy. We measured the distribution of leukocyte subsets and their relation to markers of inflammatory activation in patients with CHF. We also assessed the impact of treatment with β-blockers, angiotensin-converting enzyme (ACE) inhibitors, angiotensin receptor blockers (ARB), aldosterone antagonists, and statins.

## Materials and Methods

### Study population

We prospectively studied 75 patients with CHF who were recruited from the Royal Brompton Hospital outpatients' department between April 2004 and September 2005. The diagnosis of CHF was based on symptoms, clinical signs, and documented left ventricular impairment measured by echocardiography (left ventricular ejection fraction≤45%). All patients were on unchanged medication for at least four weeks. At the time of assessment no patients were taking non-steroidal anti-inflammatory drugs or steroid hormones. Subjects with clinical signs of infection, rheumatoid arthritis, or cancer were excluded. In addition, we enrolled 20 age-matched healthy control subjects (patients' relatives and hospital staff). At the time of assessment none of the control subjects were taking any medication. The ethics committee on human research of the Royal Brompton Hospital, London, UK, approved the study, and all subjects provided written informed consent. The study was performed in adherence to the Declaration of Helsinki.

### Fluorescence activated cell sorting (FACS) analysis

Venous EDTA blood was drawn after 15 minutes of semi-supine rest in the morning. Samples of 100 µl were incubated for 25 minutes at +4°C in the dark with the respective antibody combinations. Red blood cells were lysed using FACS lysing solution (Becton Dickinson, Oxford, UK) according to the manufacturer's instructions. Cells were then washed using phosphate buffered saline (PBS), supplemented with 2% fetal calf serum and 0.01% sodium azide (all from Sigma-Aldrich, Poole, UK). 30,000 cells were analysed per sample using a FACSort^®^ and CellQuest^®^ software (both from Becton Dickinson). The following antibodies (with the respective isotype controls) were used in the following combinations: (i) fluorescein isothiocyanate (FITC) labelled monoclonal mouse-anti-human CD4 IgG_1_ antibody (Ab) (Sigma-Aldrich)/R-phycoerythrin (PE) labelled monoclonal mouse-anti-human CD8 IgG_2a_ Ab (Sigma-Aldrich)/Peridinin-chlorophyll-protein Complex (PerCP) labelled monoclonal mouse-anti-human CD3 IgG_1_ Ab (Becton Dickinson), (ii) FITC labelled monoclonal mouse-anti-human CD14 IgG_2a_ Ab (Sigma-Aldrich)/PE labelled monoclonal mouse-anti-human CD19 IgG_1_ Ab (Sigma-Aldrich), (iii) PE labelled monoclonal mouse-anti-human HLA-DR IgG_2a_ Ab (BD PharMingen). In addition, an untreated (*i.e.* no Ab staining) sample was analysed for each patient.

### Whole blood culture and lipopolysaccharide treatment

A substudy was performed in 42 patients with CHF and 11 healthy controls of the above described cohort. Venous citrated blood was drawn as described above. Whole blood samples were diluted 1∶1 with RPMI 1640 (Life Technologies Ltd., Paisley, UK) supplemented with 10 U/mL heparin (Leo Laboratories Ltd., Bucks, UK). Afterwards, 1 mL aliquots were placed in 1.5 mL Eppendorf tubes (Eppendorf UK Ltd., Cambridge, UK). E. coli-derived LPS (serotype 0111:B4, Sigma-Aldrich Co. Ltd., Irvine, U.K.) was added to achieve a final concentration of 0.1, 1, 10, or 100 ng/mL. LPS was diluted in RPMI 1640. The addition of RPMI 1640 alone served as a control. Dilutions, aliquoting and stimulations were carried out under sterile conditions. Following the addition of endotoxin (lipopolysaccharides, LPS), all samples were incubated for 6 hours in a humidified atmosphere (37°C, 5% CO_2_). Pilot experiments had demonstrated a maximal TNFα secretion after 6 hours incubation (data not shown). Upon centrifugation, supernatants were harvested and frozen immediately at −80°C for later analysis. Cell viability was>90% as assessed using trypan blue exclusion.

### Detection of TNFα and IL-6 from culture supernatant

The detection of TNFα and IL-6 was performed using standard enzyme-linked immunosorbent assay (ELISA) kits according to the manufacturer's instructions (R & D Systems, Minneapolis, USA). The detection limit was 15 pg/mL. All samples were frozen at −80°C until analysis.

### Detection of plasma cytokines and cytokine receptors

Serum levels of TNFα, soluble TNF receptor-1 and 2 (sTNFR-1 and sTNFR-2), IL-6, and IL-1β were determined using the respective Quantikine HS immunoassay kits (all from R&D, Minneapolis, USA). The lower limits of detection are 0.106 pg/mL, 7.8 pg/mL, 7.8 pg/mL, 0.039 pg/mL, and 0.057 pg/mL, respectively.

### Statistical analysis

Data are presented as mean±SD. All data were checked for normal distribution using the Kolmogorov-Smirnov test. Non-normally distributed variables were log-transformed to achieve normal distribution before further analysis. Analysis of variance (ANOVA) with Fisher's post hoc test, repeated measures ANOVA, Student's unpaired and paired *t*-tests, simple regression, and the chi-square test were used as appropriate. The effect of beta-blocker therapy was assessed using the ANOVA method of testing. A *p*-value<0.05 was considered significant.

## Results

### Study population

There were no significant differences between healthy control subjects and patients with CHF in terms of age, sex, weight, number of smokers, or body mass index. As expected there were differences in standard biochemistry (Table 1). Patients with CHF presented with significantly lower total cholesterol (p<0.0001) and high density lipoprotein cholesterol levels (p = 0.0003). They had lower hemoglobin (p = 0.02) and higher creatinine and uric acid levels (both p<0.0001 *vs*. control, Table 1). CHF patients (n = 75) were on standard medication for their condition with diuretics (95%), angiotensin converting enzyme (ACE) inhibitors (71%), angiotensin receptor blockers (ARB) (28%), β-blockers (68%), aldosterone antagonists (57%), and statins (64%). 51 CHF patients received beta-blockers, and the following beta-blockers were prescribed: 39% bisoprolol (n = 20), 59% carvedilol (n = 30), 2% metoprolol (n = 1). Five CHF patients (2 in the group receiving β-blockers) were not being treated with either an ACE-inhibitor or an ARB. Apart from β-blockers, statistical differences in regard to the distribution of the patients' medication were not observed (beta-blocker naïve vs. beta-blocker treated patients, n.s. for all comparisons). A total of 16 patients with CHF had died before December 2007 when the database was censored.

**Table 1:** Baseline data for control subjects and patients with CHF.

	Controls	CHF Patients	p-Value
Number (female)	20 (8)	75 (16)	0.09
NYHA class		2.5±0.7	
Aetiology (ischemic/non-ischemic, %)		65/35	
LVEF (%)		31.9±11.1	
Age (yr)	63.3±10.1	67.8±10.7	0.09
BMI (kg/m^2^)	26.4±3.0	28.9±5.4	0.26
Hemoglobin (g/dL)	14.5±1.1	13.5±1.7	**0.02**
Sodium (mmol/L)	139±2	137±4	**0.01**
Potassium (mmol/L)	4.3±0.3	4.3±0.5	0.4
Creatinine (µmol/L)	83±15	124±44	**<0.0001**
Alkaline phosphatase (U/L)	66±17	76±33	0.18
Aspartate transaminase (U/L)	28±16	23±10	0.18
Gamma glutamyl transpeptidase (U/L)	34±26	55±48	**0.03**
Uric Acid (µmol/L)	321±79	456±130	**<0.0001**
Cholesterol (mmol/L)	6.0±0.7	4.7±1.2	**<0.0001**
High density lipoprotein (mmol/L)	1.5±0.4	1.1±0.3	**0.0003**

### LPS stimulations and serum cytokine levels

**Figure 1:**
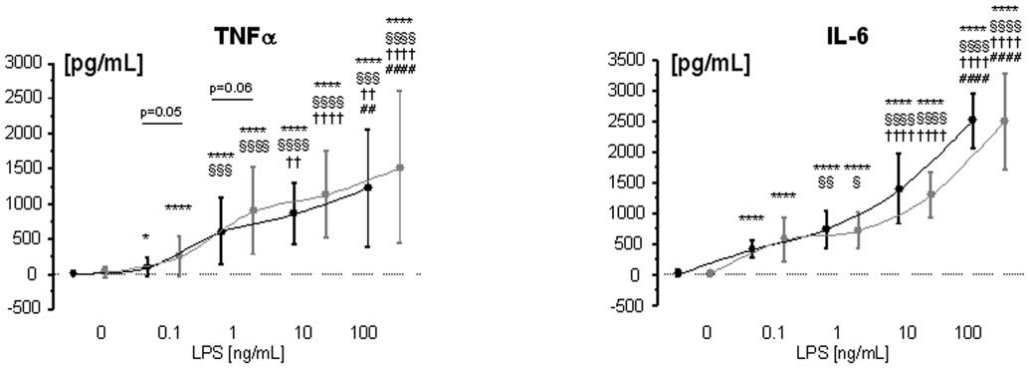
LPS-stimulated production of TNFα (panel A) and IL-6 from whole blood from healthy control subjects (n = 11, black line) and patients with CHF (n = 42, gray line). * *vs.* unstimulated control (0 ng/mL LPS); § *vs.* 0.1 ng/mL LPS; † *vs.* 1 ng/mL LPS; # *vs.* 10 ng/mL LPS. One symbol denotes p<0.05, two symbols p<0.01, three symbols p<0.001, four symbols p<0.0001.

Whole blood from both healthy subjects and patients with CHF produced substantial levels of TNFα and IL-6 when stimulated with 0.1, 1, 10, or 100 ng/mL LPS (Figure 1). There was a trend towards higher LPS-stimulated TNFα production levels in patients with CHF than in healthy control subjects at two LPS concentrations, 0.1 (p = 0.05) and 1 ng/mL (p = 0.06). There was no significant difference with regards to the serum concentrations of C-reactive protein, IL-1β, and TNFα (all p>0.25, Table 2). There were significantly higher levels of IL-6 (p<0.0001), soluble TNFα receptor (sTNFR) 1 (p = 0.0025), and sTNFR-2 (p = 0.0007, Table 2).

**Table 2:** Baseline marker of immune activation and cytokines.

	Control Subjects	CHF Patients	p-Value
White Blood Cells (×10^9^/L)	7.0±1.6	7.5±2.1	0.39
CRP (mg/L)	8.3±5.6	10.1±7.5	0.28
IL-1β (pg/mL)	1.1±1.7	1.2±3.0	0.96
IL-6 (pg/mL)	2.2±2.5	4.9±4.2	**<0.0001**
TNFα (pg/mL)	4.2±8.4	2.0±2.4	0.43
TNFR-1 (pg/mL)	1252±319	2086±1082	**0.0025**
TNFR-2 (pg/mL)	1591±466	2780±1360	**0.0007**

### Distribution of leukocyte subsets

**Figure 2:**
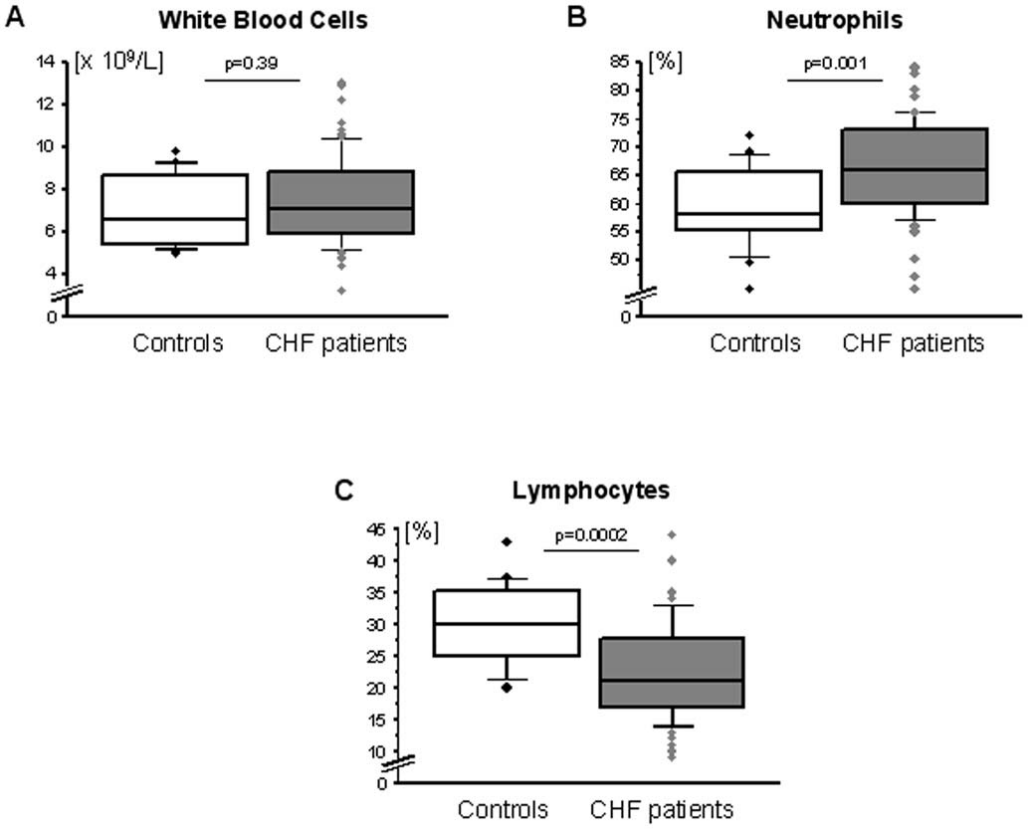
Distribution of leukocytes (panel A) and their subsets (panels B and C) in healthy control subjects (n = 20) and patients with CHF (n = 75).

**Figure 3:**
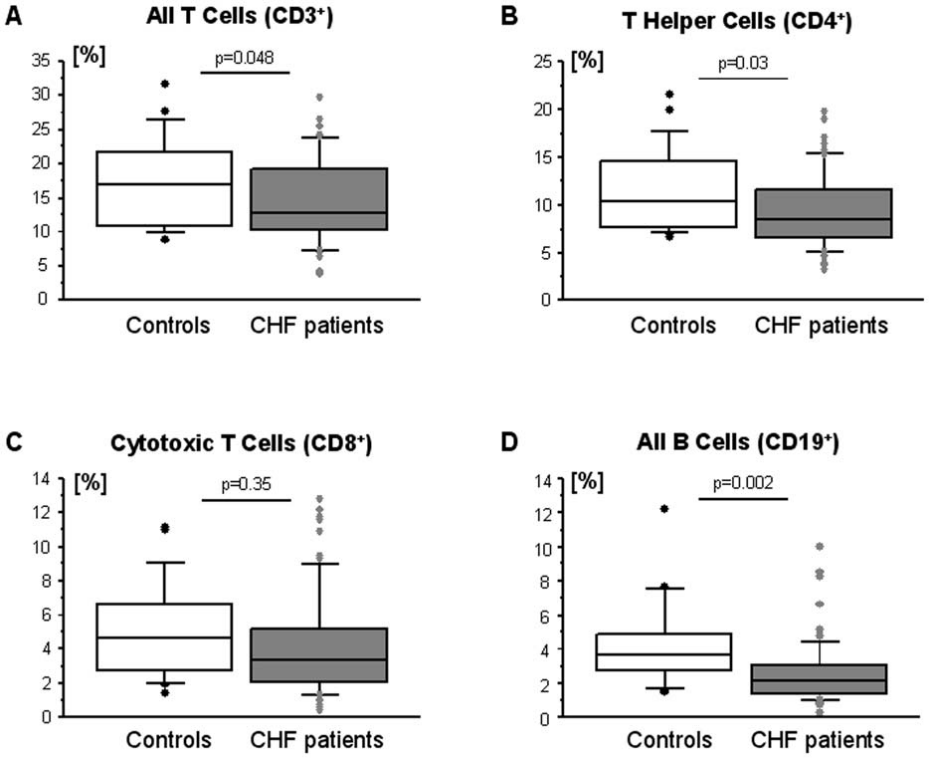
Distribution of lymphocyte subsets in healthy control subjects (n = 20) and patients with CHF (n = 75).

There were no significant differences in the absolute number of white blood cells between healthy control subjects and patients with CHF (p = 0.39, Figure 2A). No differences in either the absolute or in the relative numbers were noted for monocytes (control: 0.35±0.09×10^9^/L or 4.9±1.1%; CHF: 0.38±0.12×10^9^/L or 5.2±1.6%, both p>0.3), eosinophils (control: 0.23±0.09×10^9^/L or 3.1±1.5%; CHF: 0.26±0.17×10^9^/L or 3.5±2.2%, both p>0.4), or basophils (control: 0.08±0.04×10^9^/L or 0.9±0.5%; CHF: 0.07±0.05×10^9^/L or 0.8±0.4%, both p>0.2). However, both the absolute and the relative number of neutrophils were increased in patients with CHF (control: 4.2±1.2×10^9^/L; CHF: 5.0±1.6×10^9^/L, p = 0.048, Figure 2B). Additionally, the absolute and the relative number of lymphocytes was decreased in these patients (control: 2.1±0.6×10^9^/L; CHF: 1.7±0.7×10^9^/L, p = 0.019, Figure 2C). The distribution of lymphocyte subsets in healthy control subjects and patients with CHF is given (Figure 3).

### Correlation analysis

Significant negative correlations existed between serum levels of sTNFR-1 and the relative number of lymphocytes, CD3^+^ T cells, T helper cells, B cells, and HLA-DR expressing lymphocytes (Table 3). Similarly, there was a negative correlation between sTNFR-2 and the relative number of lymphocytes and HLA-DR expressing lymphocytes (Table 3). There was a trend towards a negative correlation between sTNFR-2 and T helper cells (p = 0.06) and B cells (p = 0.08). Similar observations were made for IL-6, but not for IL-1β, TNFα, and CRP (Table 3). Significant positive correlations were detected between serum creatinine levels and sTNFR-1, sTNFR-2, and IL-6.

**Table 3:** Table Regression.

	IL-1β (pg/mL)	IL-6 (pg/mL)	TNFα (pg/mL)	TNFR-1(pg/mL)	TNFR-2 (pg/mL)	LPS-stimulated TNFα (pg/mL)	CRP (mg/L)
**Lymphocytes (%)**	NS	r = −0.242	NS	**r = −0.345**	**r = −0.287**	NS	NS
		p = 0.059		**p = 0.006**	**p = 0.024**		
**CD3^+^ T cells (%)**	NS	**r = −0.30**	NS	**r = −0.371**	r = −0.188	NS	NS
		**p = 0.022**		**p = 0.004**	p = 0.158		
**CD4^+^ T helper cells (%)**	NS	NS	NS	**r = −0.368**	r = −0.245	NS	NS
				**p = 0.005**	p = 0.063		
**CD8^+^ cytotoxic T cells (%)**	NS	r = −0.239	NS	r = −0.245	NS	NS	NS
		p = 0.071		p = 0.064			
**CD19^+^ B cells (%)**	NS	NS	r = −0.224	**r = −0.282**	r = −0.224	NS	NS
			p = 0.08	**p = 0.028**	p = 0.082		
**HLA-DR^+^ lymphocytes (%)**	NS	r = −0.244	r = −0.183	**r = −0.38**	**r = −0.3**	NS	NS
		p = 0.062	p = 0.165	**p = 0.003**	**p = 0.021**		
**Neutrophils (%)**	r = +0.169	r = +0.219	NS	r = +0.217	r = +0.188	NS	NS
	p = 0.189	p = 0.087		p = 0.09	p = 0.144		
**Monocytes (%)**	NS	NS	r = +0.226	r = +0.201	r = +0.217	r = +0.206	NS
			p = 0.077	p = 0.118	p = 0.091	p = 0.095	
**Creatinine (µmol/L)**	NS	**r = +0.307**	r = +0.224	**r = +0.637**	**r = +0.643**	NS	NS
		**p = 0.015**	p = 0.08	**p<0.0001**	**p<0.0001**		

NS = non-significant with p>0.2.

### Effect of patients' medication

We tested the effects of the patients' medication with angiotensin converting enzyme inhibitors/angiotensin receptor blockers, β-blockers, aldosterone antagonists, and statins on lymphocyte and neutrophil distribution, because these leukocyte populations were significantly different between healthy subjects and patients with CHF. Whilst beta-blockers had significant effects on the distribution pattern, no such effect was noted with angiotensin converting enzyme inhibitors/angiotensin receptor blockers, aldosterone antagonists, or statins (all p>0.18). The results for β-blockers are presented in Table 4.

**Table 4:** Effect of β-blockers.

	Control Subjects (n = 20)	CHF Patients on a Beta-Blocker (n = 51)	CHF Patients without a Beta-Blocker (n = 24)	p-Value for Trend (#)
White Blood Cells (×10^9^/L)	7.0±1.6	7.5±2.1	7.4±2.2	0.67
Lymphocytes (%)	30.2±6.3	25.2±7.4 ††	16.8±5.1 †††† ****	**<0.0001**
CD3^+^ T cells (%)	17.5±6.4	16.3±5.9	10.5±4.1 ††† ***	**<0.0001**
CD4^+^ T helper cells (%)	11.6±4.3	10.6±3.8	7.0±2.7 ††† ***	**0.0001**
CD8^+^ cytotoxic T cells (%)	4.9±2.8	4.8±3.3	2.9±1.8 †† **	**0.008**
CD19^+^ B cells (%)	4.2±2.6	2.8±1.9 †††	2.1±1.7 †† *	**0.004**
Neutrophils (%)	59.3±7.2	64.0±8.4 †	71.4±6.6 †††† ***	**<0.0001**
Monocytes (%)	4.9±1.1	5.1±1.3	5.5±2.1	0.35
IL-1β (pg/mL)	1.1±1.7	0.9±1.7	1.8±4.6	0.49
IL-6 (pg/mL)	2.2±2.5	4.2±3.0 †††	6.2±5.9 ††††	**<0.0001**
TNFα (pg/mL)	4.2±8.4	2.1±2.6	1.8±2.0	0.73
TNFR-1 (pg/mL)	1252±319	1909±1076 †	2430±1032 ††† *	**0.001**
TNFR-2 (pg/mL)	1591±466	2612±1386 ††	3106±1277 †††	**0.001**

† p<0.05 vs. control; †† p<0.01 vs. control; ††† p<0.001 vs. control; †††† p<0.0001 vs. control * p<0.05 vs. patients on a beta-blocker; ** p<0.01 vs. patients on a beta-blocker; *** p<0.001 vs. patients on a beta-blocker; **** p<0.0001 vs. patients on a beta-blocker. # p-values were calculated using the analysis of variance (ANOVA) method of testing.

## Discussion

Our data show that the distribution of lymphocyte subsets and neutrophils is significantly altered in patients with CHF as compared to healthy age-matched controls. Whilst no change in the absolute number of white blood cells was noted, we found decreases in the number of lymphocytes, mainly driven by reductions in T helper cells and B cells. These effects were most pronounced in β-blocker naïve patients who also presented with lower relative numbers of cytotoxic T cells as compared to patients who were on such a drug (Table 4). Thus, it appears that β-blockers are able to reverse changes in leukocyte distribution seen in patients with CHF.

In view of these findings, one possible explanation for leukocyte redistribution, among others, is linked to elevated levels of catecholamines, which have been described by many independent researchers in patients with CHF [7], [8], [9], [10]. Since virtually all lymphoid cells express β-adrenergic receptors [2], catecholamines exert direct effects on these cells. β_2_-adrenergic receptors are most abundantly expressed, but their number and their sensitivity vary significantly between cell types [11]. Experimental catecholamine administration is known to yield two distinct responses: an early mobilization of lymphocytes that occurs within minutes is followed by an increase in granulocytes with relative lymphopenia, which occurs after a few hours [12]. The picture in our patients with CHF might represent this late phase of catecholamine exposure. Moreover, our findings are in line with those from earlier reports that demonstrated that adrenaline injection induced the most profound changes in the fraction of T- and B-lymphocytes [13], [14]. Treatment of healthy volunteers with terbutaline, a selective β_2_-agonist, has been shown to decrease the number of circulating lymphocytes mainly in those subsets with the greatest β-adrenergic sensitivity *in vitro* [15]. This effect has been ascribed to the inhibition of lymphocyte proliferation [16]. Most catecholamine-induced effects appear to be mediated via β_2_-adrenergic receptors but, it has also been reported that increases in granulocytes involve β-adrenergic receptor stimulation [12]. Thus, distinct mechanisms lead to the recruitment of different immune cells. From a teleological point of view, it has been argued that it is essential for survival that acute stress (which triggers catecholamine release) induces a recruitment of cells active in the first line of immunologic defence, *i.e.* an early increase in non-specific granulocytes [12]. However, we did not measure catecholamine levels as this was beyond the scope of this study. Nevertheless, this is a limitation of the study and catecholamine levels should be assessed in future analyses.

Alternatively, higher cortisol levels may be responsible for the observed effects in patients with CHF. Cortisol may induce granulocytosis and relative lymphopenia. Moreover, disturbed intestinal microcirculation and barrier function in CHF may induce a state of chronic endotoxinemia which may yield lymphopenia.

Nevertheless, a vast array of factors contributes to the release of catecholamines in patients with CHF, and one important factor is the influence of pro-inflammatory cytokines. It was shown decades ago that IL-1, IL-6, and TNFα can trigger the activation of the sympathetic nervous system in the brain [2]. Peripheral injection of IL-1β, for example, produces a long-lasting increase in the activity of the sympathetic nerves of the spleen, which in turn yields an increase in the release of noradrenaline from this organ [17]. Pro-inflammatory cytokines are activated earlier in the course of CHF than the classic neurohormones like angiotensin II or noradrenaline [18], [19]. This buttresses their preeminent role in the progression of CHF. We found significant correlations between most lymphocyte subsets that we investigated and sTNFR-1, sTNFR-2 but not TNFα itself (Table 3). This finding is in line with earlier reports, which found that plasma concentrations of soluble TNFα receptors vary less than those of TNFα [20]. TNFα has also been shown to provoke renal dysfunction, which is the most likely explanation for the association between creatinine and the plasma levels of both soluble TNFα receptors (Table 3) [21].

Some limitations of this analysis demand further discussion, among these the fact that the number of study patients is rather limited. Moreover, our results may be influenced by the fact that the patients not receiving β-blockers might have been sicker which may have led physicians not to prescribe β-blockers as eg. they had a lower blood pressure. Furthermore, as stated before, we did not assess catecholamine levels. As chronic lymphopenia may theoretically yield an increased rate of infection, it is tempting to speculate that the mortality benefit provided by the use of β-blockers in CHF may in part be explained by the effects observed here. This, however, needs to be interpreted with caution. The results presented here need to be confirmed in a larger analysis.

In conclusion, we found a significant redistribution of leukocyte subsets with an increase in granulocytes and relative lymphopenia in patients with stable CHF. This finding is in line with previous studies [18], [22] that were performed before β-blockers were implemented into the treatment guidelines of CHF. An association of β-blockers with leukocyte redistribution was found and it may be speculated that beta-blockers partly reverse the CHF-associated leukocyte redistribution. Elevated levels of pro-inflammatory cytokines may be responsible for this phenomenon, because they are known to trigger catecholamine-release via stimulation of the sympathetic nervous system in the brain.
